# Flame Retardant from Eugenol as Green Modifier for Epoxy Resins

**DOI:** 10.3390/ijms26125861

**Published:** 2025-06-19

**Authors:** Danuta Matykiewicz, Beata Dudziec, Sławomir Michałowski

**Affiliations:** 1 Faculty of Mechanical Engineering, Poznan University of Technology, Piotrowo 3, 61-138 Poznan, Poland; 2 Faculty of Chemistry and Center for Advanced Technologies, Adam Mickiewicz University in Poznan, Uniwersytetu Poznanskiego 8 and 10, 61-614 Poznan, Poland; beata.dudziec@gmail.com; 3 Department of Chemistry and Technology of Polymers, Cracow University of Technology, Warszawska 24, 31-155 Cracow, Poland; slawomir.michalowski@pk.edu.pl

**Keywords:** flame retardant, eugenol, green epoxy resin

## Abstract

A biobased flame retardant, trieugenylphosphate (TEP), was synthesized from eugenol and incorporated at concentrations of 10 and 30 wt.% into the epoxy matrix. Flammability and thermal stability were investigated using the UL-94 test, pyrolysis–combustion flow calorimetry (PCFC), and thermogravimetric analysis (TGA). Thermal and thermomechanical properties were examined by differential scanning calorimetry (DSC) and dynamical mechanical thermal analysis (DMTA). The modified resin with TEP content showed self-extinguishing properties and acceptable thermal and thermomechanical properties. Furthermore, the microcalorimetric method proved that the introduction of the TEP additive to the epoxy matrix reduced the values of p_c_HRR (414.4 ± 5.5 W/g), THR (29.1 ± 0.6 kJ/g), and HRC (446 ± 7 J/g·K) for the sample tested compared to the unmodified resin.

## 1. Introduction

To ensure fire safety, structural and coating materials made of epoxy resins are modified with various reactive or non-reactive flame-retardant additives. Eugenol has wide industrial applications in pharmacy, cosmeceutics, dentistry, and food flavoring [1]. The chemical structure of this phenol allows easy modification to obtain a wide range of monomers of biological origin [2]. Recently, it has been the focus of attention due to its great potential for combining with other compounds and modifying polymer compounds [3]. The fire resistance of epoxy resins can be achieved by methods including the introduction of internal flame retardants, additive flame retardants, and reactive flame retardants [4].

The polymer combustion process is a complex combination of energy feedback from the flame to the polymer surface with gasification of the polymer and the generation of flammable degradation products [5]. The main purpose of flame retardants is to reduce the risk of ignition of polymer substrates by reducing the rate of spread of flame during a fire [6]. Halogen-free flame retardants commonly used for epoxies include phosphorus flame retardants, carbon-based materials, silicon flame retardants, inorganic nanofillers, and metal-containing compounds [7]. Organophosphorus compounds commonly decompose during thermal degradation of the polymer matrix to generate polyphosphoric acid, which can react with the polymer and help form char residues [8]. The mode of action for phosphorus flame retardants depends on the degree of phosphorus oxygenation. In alkyl phosphates, elimination occurs rapidly at low temperatures, whereas in aryl phosphates, it takes place at slightly higher temperatures. In contrast, in phosphonate and phosphinate, the elimination of phosphoric acid proceeds more slowly and requires higher temperatures. The acids formed during this process subsequently undergo further degradation, leading to the formation of volatile compounds [9]. In recent years, the possibilities of using modified plant raw materials with phosphorus to produce materials with increased fire resistance and flame retardants have gained focus [10,11].

Biomass compounds such as resveratrol [12], lignin [13], and vanillin [14] have been successfully used in recent years to obtain new flame retardants due to their environmental friendliness and sustainability. Resveratrol-based epoxy resin (REEP) with low flammability possessing trifunctional epoxy groups and rigid conjugate structures and cured by methyl hexahydrophthalic anhydride (MeHHPA) has been obtained. A vanillin-derived bisDOPO co-curing agent for epoxy resin capable of improving flame retardancy, mechanical strength, and transparency has been developed [14]. A biobased hyperbranched polymer flame retardant has been obtained from quercetin and phenylphosphoryl dichloride [15]. A latent curing agent derived from biomass has been produced by neutralization between imidazole and phytic acid in water at room temperature and the introduction of epoxy resin [16]. Biobased flame retardants are sustainable additives introduced into epoxy resins derived from renewable resources such as plants [17]. They can be obtained by modifying the chemical structure of natural compounds or by directly using virgin natural materials [18,19]. A biobased flame retardant containing phosphaphenanthrene groups containing triscardanyl phosphate has been obtained from cardanol via dehydrochlorination, epoxidation, and the ring-opening reaction [11]. The modification of wheat starch, wheat protein, xylan, and tannin with phosphate salts in molten urea has been utilized to obtain environmentally friendly flame retardants [20]. Furthermore, a biobased flame retardant based on eugenol (DPSi-ED) and dihydro-9-oxa-10-phosphaphenanthrene-10-oxide (DOPO) has been produced [21]. An eugenol-containing phosphorus–silicone hybrid compound (EGDS-DOPO) may be used to improve flame retardancy and enhance the mechanical performance of epoxy/amine materials [22]. The inclusion of phosphorus as a linker between phenol units has been shown to confer flame retardant properties to epoxy resin cured with meta-xylylene diamine, without the use of flame-retardant additives [23]. Unfortunately, many of the biobased agents currently being described are not commercially available at suitable prices for the chemical industry [24].

The preparation of flame-retardant epoxy resins with good mechanical properties from biobased materials is still a major challenge. Therefore, the impact of a flame retardant obtained from eugenol on the properties of epoxy resins has been assessed. The UL-94 flammability test and pyrolysis–combustion flow calorimetry (PCFC) were used to assess the effectiveness of the flame retardant. Thermal stability was evaluated using thermogravimetric analysis (TGA). Thermal and thermomechanical properties were investigated using differential scanning calorimetry (DSC) and dynamic mechanical thermal analysis (DMTA). A previously undescribed application of a biobased flame retardant for the modification of epoxy resin has been provided.

## 2. Results

A biobased flame retardant, trieugenylphosphate (TEP), was synthesized from eugenol and incorporated at concentrations of 10 and 30 wt.% into the unmodified epoxy resin based on Diglycidyl ether of bisphenol A (DGEBA).

### 2.1. Fourier Transform Infrared Spectroscopy

**Figure 1:**
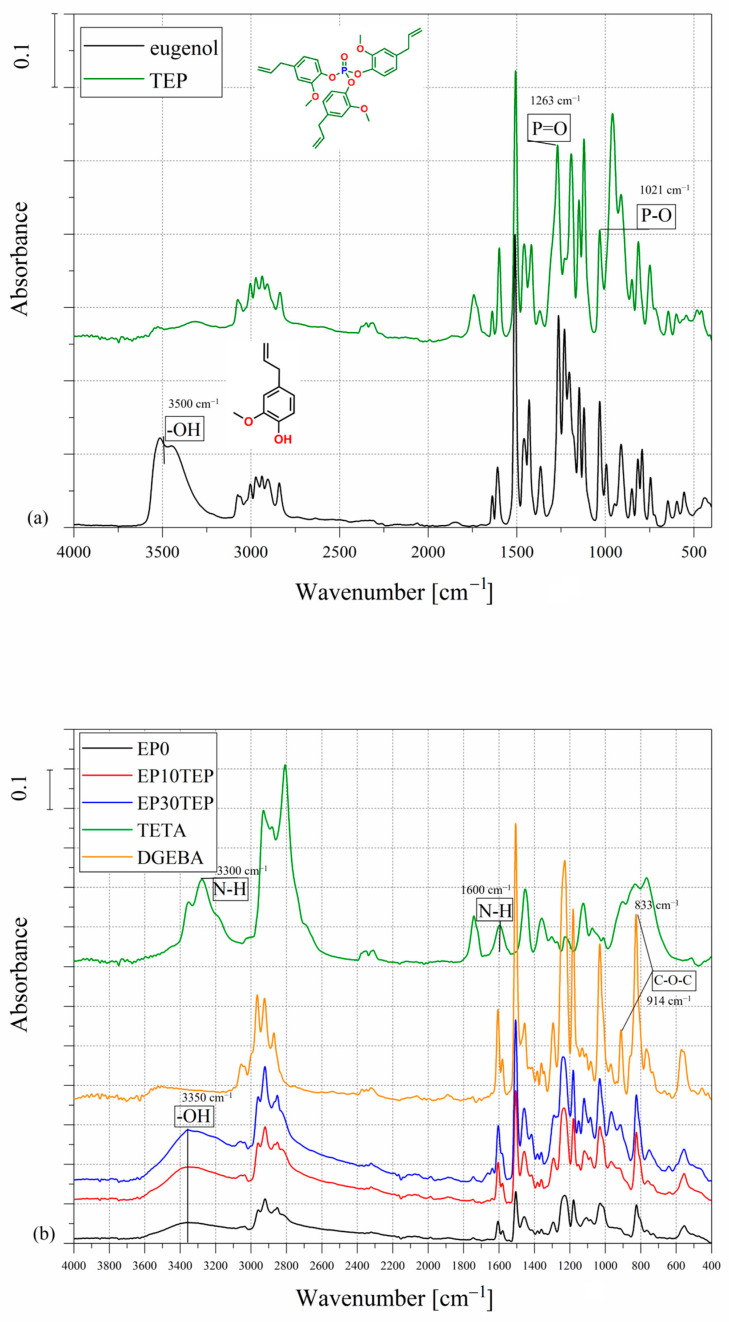
FTIR spectra of (**a**) eugenol and trieugenylphosphate (TEP) and (**b**) cured samples EP0, EP10TEP, EP30TEP, uncured epoxy monomer (DGEBA), and curing agent (TETA).

The FTIR method was used to analyze the chemical structure of trieugenylphosphate and the cured epoxy casts. Figure 1a shows the spectra of the natural biophenol–eugenol and the obtained trieugenylphosphate. Peaks were observed for eugenol at 720−1250 cm^−1^ from the vC=C bond, at 1600 and 1500 cm^−1^ attributed to the stretching of C=C of the aromatic group, at 2700−2900 cm^−1^ from CH_3_ groups, and at 3500 cm^−1^ from hydroxyl groups. The disappearance of characteristic peaks at 3500 cm^−1^ for the hydroxyl group from eugenol was observed for the TEP sample. The successful eugenol substitution reaction was confirmed, as well as new P=O and P−O bands recorded at 1263 and 1021 cm^−1^.

During the curing reaction in the first stage, the amine reacts with the epoxy group and a hydroxyl group forms (Figure 1b). All cured casts displayed a broad band at 3200−3600 cm^−1^, attributed to −OH stretching, suggesting ring opening of the epoxy groups. Furthermore, the peaks of the epoxy ring were not recorded at 914 and 833 cm^−1^, which would indicate unreacted monomer groups. Moreover, for EP10TEP and EP30TEP samples, intense peaks were observed at 1510−1615 cm^−1^ from aromatic stretching C=C and at 1263 and 1021 cm^−1^ from P=O and P−O bonds, respectively. For the TETA curing agent, N–H stretching and deformation peaks were observed in the wavelength range of 3200–3400 cm^−1^ and 1650–1500 cm^−1^, respectively.

### 2.2. UL-94 Vertical and Horizontal Burning Test

**Figure 2:**
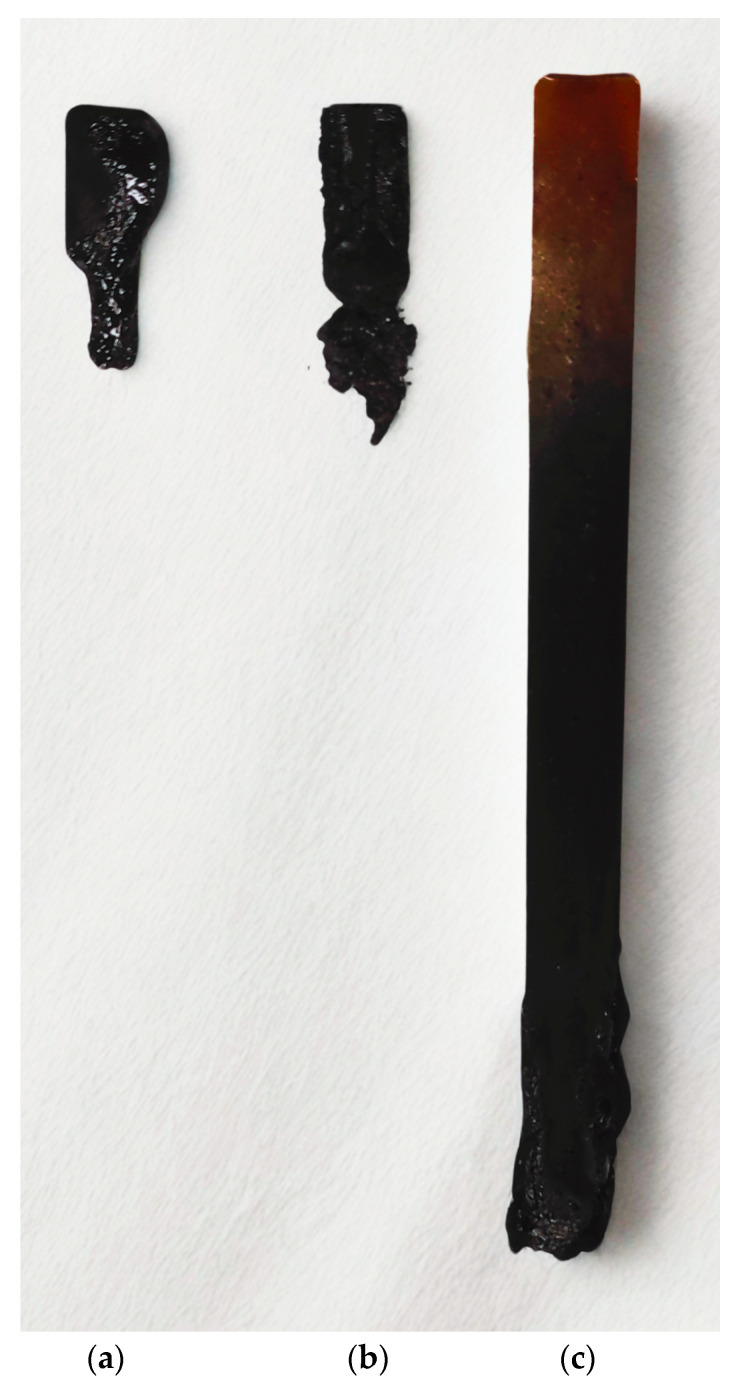
The samples tested after the UL-94 vertical burning tests: (**a**) EP0, (**b**) EP10TEP, (**c**) EP30TEP.

Halogen-based flame retardants are being phased out because they cause serious environmental problems, i.e., they emit toxic and corrosive fumes during combustion. Therefore, the search for alternative compounds is of key importance for applications such as the electronic and construction industry [25,26]. Flammability testing protocols are the foundation for product safety and compliance. Flame spread describes how a fire spreads across the surface of a material; fireproof materials should not spread flame. The UL method allows one to assess whether a material is self-extinguishing and whether burning or falling droplets appear when plastics are burnt [27]. In the vertical UL-94 test, epoxy resin with 30 wt.% TEP was self-extinguishing and did not sustain combustion. EP10TEP samples were completely charred up to the handle, but no dripping was observed. In the case of unmodified EP resin, the combustion process was intense, and the dripping drops ignited the cotton wool. Sample EP 30 TEP achieved level V1. Samples EP 10 TEP and EP0 did not achieve results for classification in the vertical burning test (Table 1). The appearance of the samples after the test is shown in Figure 2.

**Figure 3:**
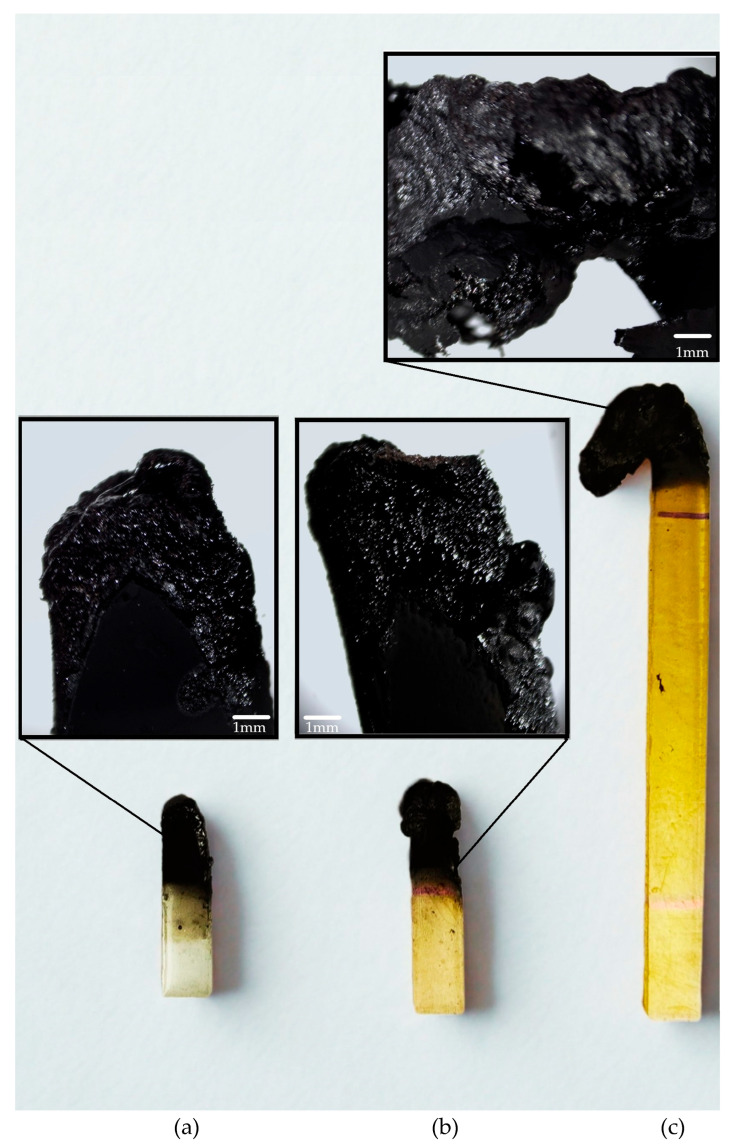
The samples tested after the horizontal burning test: (**a**) EP0, (**b**) EP10TEP, (**c**) EP30TEP.

Table 2 shows the results of the UL-94 HB horizontal burning. It should be emphasized that for the sample containing 30% TEP by mass, self-extinguishing properties were obtained. The tested sample EP30TEP was extinguished before the measurement began, which is also shown in Figure 3. Polymer combustion occurs in multiple stages, including heat, pyrolysis, ignition, and combustion [28]. As the polymer heats up, its structure begins to decompose, releasing the following products: flammable gases, molten liquid produced by the thermal decomposition of polymers, non-flammable gases, fine particulate matter composed of polymer fragments, and carbon residues [29]. The amount of ash visible in the photo, which was produced on the surface of the tested epoxy sample as a result of TEP introduction into it, significantly contributed to inhibiting the spread of fire and further degradation of the material.

### 2.3. Microcalorimetry

**Figure 4:**
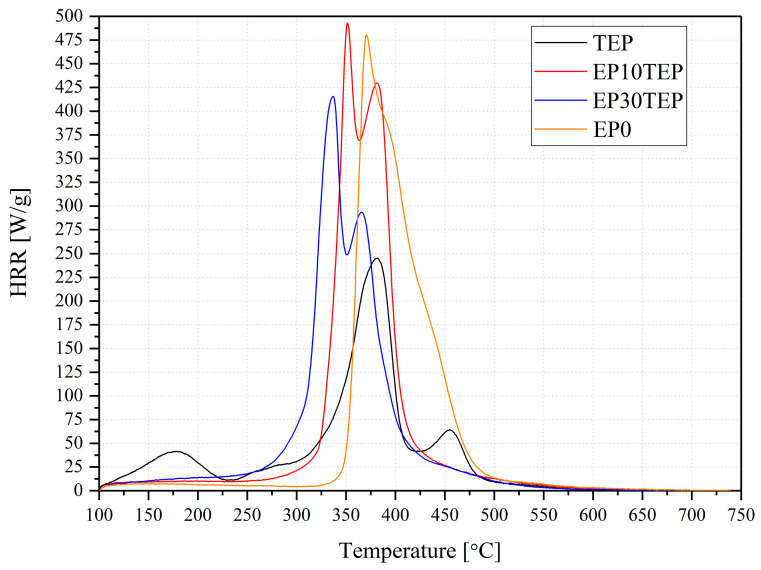
Heat release rate (HRR) as a function of temperature: TEP, EP0, EP10TEP, EP30TEP.

The data recorded are collected in Table 3 and presented in Figure 4. The heat release rate (HRR) is a crucial parameter that allows the prediction of the reaction to material fire and controlling fire hazards [30]. The heat release rate is the mass loss rate of the material multiplied by its heat of combustion. When the material reaches the maximum heat release rate (pHRR), the HRR gradually decreases with time. This is mainly due to the coating of the material with a char layer that protects the material and slows down its decomposition [31].

The results obtained confirm that the addition of TEP to the epoxy matrix slowed down the release of gases and reduced the thermal conductivity of the heat flow from the fire to the internal matrix. This phenomenon may be due to the carbon layer that was formed on the surface of the tested samples. Therefore, the low heat release capacity (HRC) values recorded for the EP30TEP samples (446 ± 7 J/g·K) were recorded compared to the commercial epoxy samples (520 ± 3 J/g·K). The carbon layer acts as an insulator against thermal radiation and prevents flammable gases from entering the fire [32,33]. The lowest p_c_HRR values were recorded for the liquid TEP compound, followed by EP30TEP, averaging 262 W/g and 414 W/g, respectively. Furthermore, the rate of heat release is influenced by various effects such as changes in the surface emissivity of the material during its decomposition, the specific heat of the reaction gases, which may have a cooling effect, and changes in the heat flux and oxygen content of the fire [34,35]. In addition, the amount of heat a material releases in a fire is described as the THR factor. The introduction of flame retardants to the polymer should decrease this value. The THR values recorded for the samples were 24.8 kJ/g for TEP, 30.9 kJ/g for EP10TEP, and 29.1 kJ/g for EP30TEP and were lower compared to the unmodified resin EP0 (32.3 kJ/g).

Two distinct peaks are visible on the HRR curves for modified samples p_1_HRR = 351 °C and p_2_HRR = 379 °C for EP10TEP and p_1_HRR = 337 °C and p_2_HRR = 363 °C for EP30TEP. In comparison, the unmodified sample showed only one distinct peak at 373 °C. TEP showed three-step decomposition at temperatures of p_1_HRR = 178 °C, p_2_HRR = 374 °C, and p_3_HRR = 447 °C. The peak at this temperature may be related to the flame retardancy of the material by the presence of a flame retardant in the gas phase. This may affect the shift of the first HRR peaks for EP10TEP and EP30TEP.

### 2.4. Thermogravimetry

**Figure 5:**
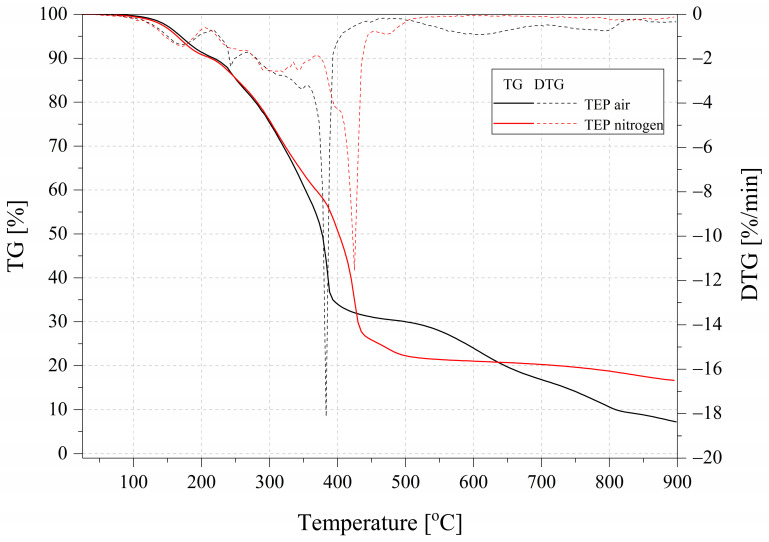
TGA and DTG curves of the TEP samples in nitrogen and air.

**Figure 6:**
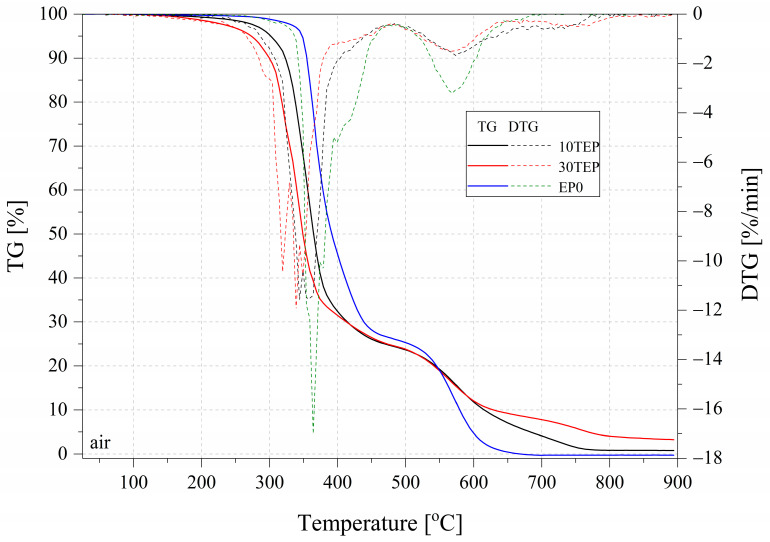
TGA and DTG curves of the tested samples in air.

**Figure 7:**
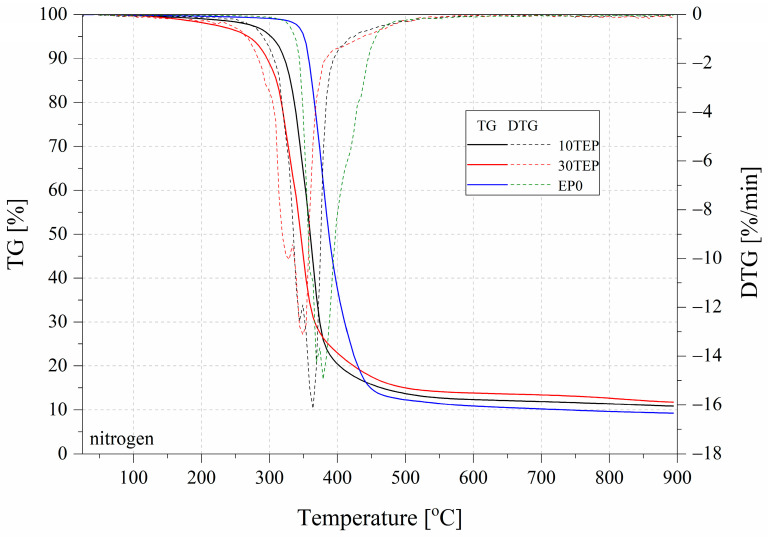
TGA and DTG curves of tested samples in nitrogen.

TGA is a technique for measuring the relationship between the mass of a substance and its temperature at a programmed controlled temperature. However, in fire hazards, materials must also be taken into account for their uncontrolled behavior in fire. Therefore, relative measurements of UL 94 and microcalorimetry constitute the completeness of these studies [36]. The results of the TGA measurement for the samples tested in both nitrogen and air are summarized in Table 4 and Figure 5, Figure 6 and Figure 7. The residual mass determined during the TGA was higher than that for the pure EP0 epoxy resin (%) and was 16.60% for TEP, 10.82% for EP10TEP, and 11.71% for EP30TEP in nitrogen. A beneficial effect that was observed after the introduction of a plant-phosphorus modifier to the epoxy matrix was a reduction in the degradation rate determined from DTG curves in both nitrogen and air. The introduction of phosphorus-based flame retardants decreased resin degradation and enhanced char formation at higher temperatures [37]. Even in the air atmosphere, a significant burn-in residue of 7.17% and 3.25% was recorded for TEP and EP30TEP samples, respectively. The introduction of the TEP additive into the epoxy matrix reduced the onset temperature of decomposition of the materials T5% and T10% compared to the unmodified resin. The main organophosphate acts as a flame retardant in the condensed or gas phase. These compounds in the condensed phase degrade at a relatively low temperature and form a protective layer of char [38].

### 2.5. Differential Scanning Calorimetry (DSC)

**Figure 8:**
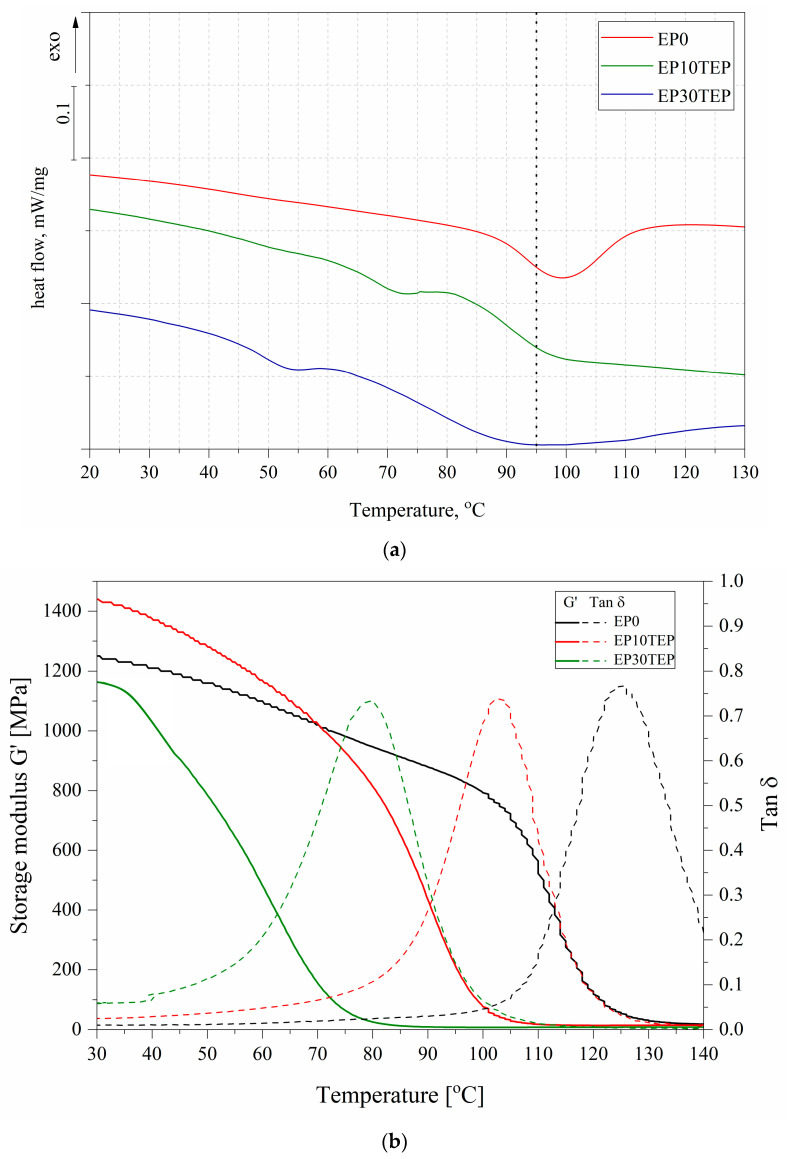
(**a**) DSC and (**b**) DMTA curves of the tested samples.

The DSC method was used to assess whether an additional post-curing reaction occurs after the curing process at elevated temperatures [39]. No additional exothermic peak was observed in the recorded DSC curves, which could indicate the occurrence of a cross-linking reaction of uncured epoxy groups (Figure 8a). The glass transition temperature (T_g_) determines the scope of application of thermosetting epoxy resins; it depends largely on the cross-linking density of cross-linking and interactions between cross-linked polymer chains [40]. It can be seen that the introduction of a flame retardant (TEP) into the epoxy matrix resulted in a decrease in the glass transition temperature of the materials (Table 5). This may be due to the plasticizing effect through the presence of complex TEP molecules and increased distances between polymer chains.

### 2.6. Dynamic Mechanical Thermal Analysis (DMTA)

DMTA was performed to determine the effect of the introduction of the TEP modifier into the epoxy matrix on the viscoelastic properties of the material. Figure 8b shows the plot of the storage modulus and damping factor on the temperature. The glass transition temperatures of the same types of materials vary according to the test method [41]. In the case of the DMTA method, much larger samples are tested; therefore, the glass transition temperatures determined are often higher than those determined by the DSC method (Table 5). The single peak of the tan delta curve may confirm the good compatibility between TEP and the epoxy matrix and the absence of the phase separation effect in the investigated materials [42]. The introduction of trieugenylphosphate in the amount of 10% wt. to the epoxy resin contributed to an increase in the storage modulus (1440 MPa) of the tested materials. This may be due to the presence of rigid phenyl structures in the TEP. However, with a significant portion of this modifier in the 30% wt., a decrease in both the value of G (1160 MPa) and T_g_ (80 °C) is observed compared to the epoxy reference sample (1250 MPa and 125 °C). Despite these differences, it can be assumed that the EP30TEP sample is characterized by satisfactory thermal–mechanical properties for its intended use and additionally has self-extinguishing properties.

## 3. Discussion

The use of renewable resources and environmentally friendly synthesis methods plays a key role in minimizing the environmental footprint associated with epoxy resin production and use. Epoxy polymer materials are often highly flammable. Therefore, the development of an additive that improves their fire resistance and is at the same time environmentally friendly is still the subject of many scientific works. On the basis of the tests carried out, it was found that the addition of TEP in the epoxy matrix plays an important role in increasing the fire resistance by inducing the formation of char. This makes it possible to replace aggressive flame retardants with compounds of plant origin. To determine the exact application of TEP in an epoxy matrix for specific applications, an in-depth analysis would have to be performed, taking into account the environmental conditions and the type of fire hazard. Nevertheless, the obtained results provide a basis for further considerations and give a broader perspective on the possibilities of using eugenol derivatives on a larger scale and in areas not yet explored. The renewable nature and natural origin of the raw material eugenol, a highly reactive polyphenol, increase its attractiveness in the context of green chemistry and sustainable materials. Due to the synthetic origin of epoxy resins, widely used in the marine, nautical, aviation, and construction industries, research on the possibility of replacing part of the petrochemical matrix with a biological material seems to be crucial in the context of reducing their negative impact on the environment [43]. The most beneficial solution would be to use inherently flame-retardant biobased epoxy resins, considering both biobased epoxy monomers and biobased curing agents. However, due to the high technical and mechanical requirements for epoxy materials, this is not always possible [17].

## 4. Materials and Methods

### 4.1. Materials

**Figure 9:**
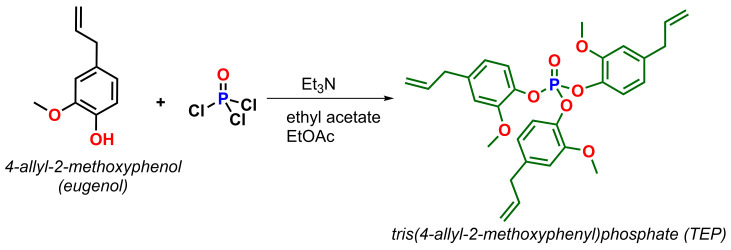
Synthesis of tris(4-allyl-2-methoxyphenyl)phosphate (TEP).

Trieugenylphosphate (TEP) was produced by the reaction according to Faye et al. when eugenol was subjected to a nucleophilic substitution reaction with phosphorus oxychloride to obtain tris(4-allyl-2-methoxyphenyl)phosphate (TEP) [44]. The detailed synthetic route of tris(4-allyl-2-methoxyphenyl)phosphate (TEP) was presented in our previous work [45]. The reaction scheme is presented in Figure 9.

Commercial unmodified epoxy resin based on Diglycidyl ether of bisphenol A (DGEBA) (Epidian 5 produced by CIECH Sarzyna S.A., Nowa Sarzyna, Poland) was selected for modification and cured with triethylenetetramine (TETA) (CIECH Sarzyna S.A., Poland), according to the proportions recommended by the manufacturer, i.e., 12 g of curing agent per 100 g of resin.

### 4.2. Sample Preparation

The TEP, epoxy resin (EP), and curing agent were stirred mechanically and then the composition was cast in a Teflon mold and cured for 24 h at ambient temperature and then post-cured at 100 °C for 1 h. Blends were made with 0, 10, and 30% by mass of the flame retardant TEP. Samples were described as EP0, EP10TEP, and EP30TEP.

### 4.3. Characterization

The UL-94 vertical (V) and horizontal burning test (HB) was carried out on samples with dimensions of 125 × 10 × 4 mm in accordance with the PN-EN 60695-11-10 standard. The burning time was measured and the dripping of the burning material was observed.

Following the above observations, the burning rate of sample *V* [mm/min] was calculated, and on this basis, the materials were classified (using the UL 94 HB classification). Materials were also classified by the UL94 V method.

The flammability tests of the samples were performed using a Pyrolysis–Combustion Flow Calorimeter (PCFC) from Fire Testing Technology Ltd. (East Grinstead, UK) according to ASTM D7309-2007. Decomposition was carried out in an inert gas atmosphere in the temperature range of 150–750 °C with a heating rate of 1 °C/s. The gases generated by pyrolysis were then oxidized in a high-temperature furnace at 900 °C for complete oxidation. The following parameters were determined: peak heat release rate (HRR) for the whole sample (p_c_HRR), peak temperature of HRR for the whole sample (Tp_c_HRR), total heat of combustion (THR), and heat release capacity (HRC).

The thermal properties of the materials were determined by thermogravimetry (TGA) in nitrogen and air atmospheres, in the temperature range from 30 to 900 °C and a heating rate of 10 °C/min (Netzsch TG 209 F1 apparatus, Selb, Germany). Samples of 10 mg were tested in ceramic vessels. The following values were determined: the temperature at which the mass loss was 10% (T10%), the residual mass at 900 °C (W%), and the maximum thermal degradation temperatures from the derived thermogravimetric graphs (DTGs).

The thermal properties of the samples were assessed by differential scanning calorimetry (DSC) using a Phoenix DSC 204 F1 Netzsch (Selb, Germany). The samples were heated from 25 to 150 °C in a nitrogen atmosphere at a heating rate of 10 °C/min. The glass transition temperature was determined from the inflection of the DSC curves.

Dynamic mechanical thermal analysis was used to define the thermomechanical properties of the samples, such as storage modulus (G’), glass transition temperature (Tg), and damping coefficient (tan δ). The measurements were performed in torsion mode (Anton Paar MCR 301,Graz, Austria) at a frequency of 1 Hz, in the temperature range from 25 to 150 °C, with a heating rate of 2 °C/min. The maximum value of tan δ was selected as the point for determining the glass transition temperature (T_g_).

## 5. Conclusions

In summary, we have shown that eugenol can be successfully used to produce biobased flame retardants for commercial resin. Obtained in the reaction of eugenol with the phosphorus compound trieugenylphosphate (TEP), due to its favorable thermal properties and liquid form, it can be easily introduced into an epoxy matrix. The modified resin with 30 wt.% TEP content showed self-extinguishing properties confirmed in the UL94 test. Furthermore, the microcalorimetric method proved that the introduction of the TEP additive to the epoxy matrix reduced the values of p_c_HRR (414.4 ± 5.5 W/g), THR (29.1 ± 0.6 kJ/g), and HRC (446 ± 7 J/g·K) for the tested sample compared to the unmodified resin for which p_c_HRR = 481.5 ± 1.1W/g; THR = 32.3 ± 0.3 kJ/g; HRC = 520 ± 3J/g·K. It can be assumed that the EP30TEP sample is characterized by satisfactory thermal–mechanical properties for its intended use and additionally has self-extinguishing properties. This confirms the possibility of using eugenol as a natural raw material for the production of flame retarders for epoxy resins. These results are important due to the assumptions of the sustainable development strategy, which in the case of chemically hardened materials is still a challenge.

## Figures and Tables

**Table 1:** UL-94 vertical burning test data.

Name	t * [s]	Classification UL94V	Falling Drops
EP	70	No Rating	yes
EP10TEP	60	No Rating	no
EP30TEP	30	V-1	no

* Total time of the first and second burning times for five specimens in the UL-94 test.

**Table 2:** UL-94 horizontal burning test data.

Name	V [mm/min]	Classification UL 94HB	Falling Drops
EP	35	HB40	yes
EP10TEP	20	HB40	no
EP30TEP	self-extinguish before measurement	HB	no

**Table 3:** Microcalorimetric data of the tested materials.

Name	p_c_HRR (W/g)	T_pc_HRR (℃)	THR (kJ/g)	HRC (J/g·K)
TEP	262.4 ± 30.0	374 ± 2	24.8 ± 2.1	283 ± 35
EP10TEP	505.2 ± 30.0	351 ± 1	30.9 ± 0.5	546 ± 35
EP30TEP	414.4 ± 5.5	336 ± 5	29.1 ± 0.6	446 ± 7
EP0	481.5 ± 1.1	373 ± 3	32.3 ± 0.3	520 ± 3

**Table 4:** Thermogravimetric data of investigated samples in an inert and air atmosphere.

Name	T5% (°C)	T10% (°C)	Residual Mass (%)	DTG Peak (°C)/max.rate (%/min)
Nitrogen				
TEP	164.5	213.7	16.60	424.4/11.61
EP10TEP	302.6	322.1	10.82	364.1/16.15
EP30TEP	268.9	296.4	11.71	346.9/13.43
EP0	351.0	357.8	9.21	376.9/16.78
Air				
TEP	169.5	218.2	7.17	384.6/21.69
EP10TEP	301.9	323.3	0.82	362.0/12.97
EP30TEP	273.3	299.7	3.25	340.7/15.53
EP0	348.2	354.7	0.10	364.7/17.75

**Table 5:** DSC and DMTA data of investigated samples.

Name	G’ at 25 °C [MPa]	tan δ	Tg [°C] from DMTA	Tg [°C] from DSC
EP0	1250	0.767	125	96
EP10TEP	1440	0.738	103	89
EP30TEP	1160	0.733	80	79

## Data Availability

Data are contained within the article and are available from the corresponding author (Danuta Matykiewicz) on request.
